# Improving Chest Monitoring through Magnetic Resonance Angiogram Image Contrast Enhancement

**DOI:** 10.3390/life13112160

**Published:** 2023-11-03

**Authors:** Beatrice Arvinti, Alexandru Isar

**Affiliations:** 1 Fundamentals of Physics for Engineers Department, “Politehnica” University Timisoara, Bd. Vasile Pârvan No. 2, 300223 Timisoara, Romania; 2 Faculty of Electronics, Telecommunications and Information Technologies, “Politehnica” University Timisoara, Bd. Vasile Pârvan No. 2, 300223 Timisoara, Romania; alexandru.isar@upt.ro

**Keywords:** angiography, filters, dual-tree discrete wavelet transform

## Abstract

Magnetic resonance angiography is a medical procedure used to offer an image of the blood vessels and organs of the body. Given the worldwide spread of cardiovascular diseases, more and more resources are invested in treating them. One of the most modern treatments involves the acquisition of images of the heart. Sometimes the contrast of these images is not satisfactory. Injecting invasive enhancement substances to obtain a better view of the cardiac route is not advisable. However, software algorithms can solve the problem. This study proposes and tests a local adaptive contrast-adjustment algorithm using the dual-tree complex wavelet transform. The method has been tested with medical data from a public database to allow comparisons to other methods. The selected algorithm further improved the contrast of images. The performances are given for evaluation, both visually (to help doctors make accurate diagnoses) and in parametric form (to show engineers which parts of the algorithm might need improvement). Compared to other contrast enhancement methods, the proposed wavelet algorithm shows good results and greater stability. Thus, we aim to avoid future pointless complications due to unnecessary contrast substances.

## 1. Introduction

Cardiac problems often arise slowly, growing more complex over time [1,2,3]. Even children have developed heart diseases at an alarming rate over the last few years [4,5,6,7]. Signs indicating cardiac dysfunction in children are bluish lips or nail beds, feeding difficulties, and trouble gaining weight. The syndromes are often observed during school: fast breathing during regular activities and tiring quickly. The most common causes of heart failure are congenital heart defects (family genetic inheritance), smoking, and diabetes. As the heart is responsible for the spreading of nutrients through the entire human body, any malfunctions should not be ignored but investigated to prevent problematic adulthood. Cardiac problems might also develop later, in the form of the following:

- Arrhythmias: breakneck heart rhythms (tachycardia) or too-slow heart rhythms (bradycardia, the main accomplice in sudden death syndrome (SDS) in newborns);
- Cardiomyopathies;
- Myocarditis in children;
- Bacterial endocarditis (due to several bacterial infections: streptococci, staphylococci and enterococci);
- Pulmonary hypertension (blood pressure in vessels leading from the heart to the lungs being too high; the patient often needs a lung transplant in this stage);
- High blood pressure (because of stressful events).

Recent findings show that the SARS-CoV-2 pandemic (COVID-19) is associated mainly with cardiac problems [8,9,10,11]. Even Kawasaki syndrome in children younger than 5 has been noticed more often after the SARS-CoV-2 pandemic [12]. Kawasaki syndrome causes the blood vessels to become inflamed and swollen, which leads to complications in supplying blood to the heart. It can be fatal in some cases (up to 3%). Left untreated, it might damage heart muscles and coronary arteries (arrhythmias and aneurysms).

This introduction does not aim to be extensive but merely to give an insight into possible cardiac malfunctions—if the symptoms are not treated. Early detection and a correct diagnosis are always better than racing to find a cure in the later stages.

**Figure 1:**
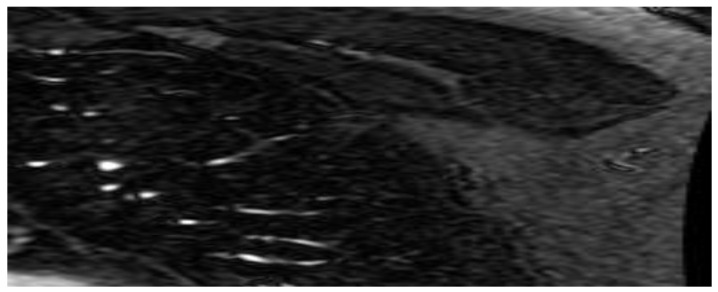
Angiogram image.

Angiograms (Figure 1) are a minimally invasive procedure: a catheter must be introduced, and a contrast solution is injected into blood vessels (arteries or veins) to provide an acceptable-quality image (map) of the blood vessels, which deliver oxygen and nutrients to all cells [13]. The reason for such a procedure is the suspicion of a problem with the blood vessels, which cannot be noticed using other techniques (such as Doppler ultrasound). Risks may increase with the patient’s condition and age. In this case, the required quality of the image is a high contrast (obtained by the contrast solution injection in most cases).

**Aim:** We aim to obtain good images with many details without injecting the contrast substance (an invasive procedure harmful to newborns [14,15], if we are studying congenital/birth-acquired abnormal heart functionalities). Because we have used magnetic resonance angiograms (MRAs) from a public database, acquired using contrast enhancers, our principal result is proof of the fact that the contrast of those images can be further improved using some image processing algorithms. We have selected a new contrast enhancement algorithm that is fast and accurate. We have compared the results obtained using the selected algorithm with the best Matlab contrast enhancement algorithm, namely CLAHE. Our objective is to apply the same algorithm to other MR images without using contrast enhancement solutions. Thus, the main aim is to make the acquisition of MR images easier, especially for pediatric patients.

## 2. Materials and Methods

Wavelets are helpful mathematical tools that represent signals in time and frequency. Time-series imaging is a technique that converts temporal data into visual representations [16,17]. 

A wavelet transform of a function *f* will be defined with the aid of a flexible function named a “mother wavelet” (MW) ψ_,_ as follows (Equation (1)):

(1) Ψf=<f,Ψa,b>=1a12·∫−∞∞f(t)·Ψt−ba¯dt,

where *a* is a parameter that describes the signal in the frequency domain and *b* is a parameter that defines the temporal localization of the signal. Any signal can be constructed through stretching and dilating the MW applied. A challenge for engineers is correctly identifying the most appropriate MW for a specific task. Research has shown that the MW’s shape should be adapted to the studied event, meaning that the MW should suit the task as best as possible [18]. This makes the choice of the optimal MW (to obtain optimal filtering results) difficult, as there are several families of MWs: orthogonal MW (of the Daubechies, Coiflet, Symmlet, Battle–Lemarié types) or biorthogonal MWs (having different filters for decomposition and reconstruction of the studied image data) [19].

Usually, the MW exposes a number of vanishing moments (VMs), which allows the choice of a better frequency or a better time localization of the data, according to necessities. Low-light and unevenly exposed images are challenging to interpret in clinical environments because of poor contrast, masked fine medical features, and high noise levels (unwanted dark spots on the angiogram).

**Figure 2:**
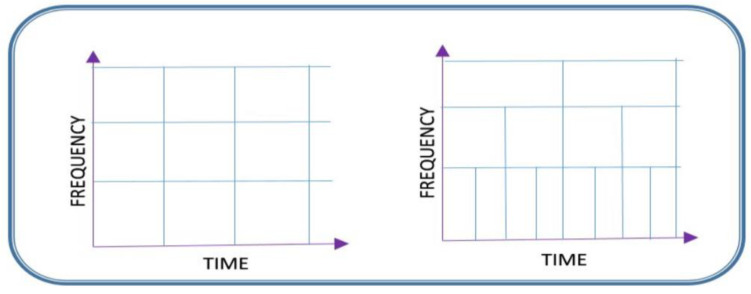
Fixed window on the left side (as in Fourier analysis) allows a poor interpretation of the studied data. Adapted-size window on the right side (as in wavelet analysis) allows a better understanding of the analyzed data at the selectivity increasing with each frequency considered.

The advantage of wavelet representations resides in their ability to give insight into the local dispersion of the coefficients, which can be better engineered by designing statistical models. They should reduce noise and prevent unnecessary noise amplification in the medical image. We can notice that the main advantage of using wavelets is their potential to analyze and correct image features considering their spatial-frequency content at different resolutions. Moving analysis windows (Figure 2) allow more selectivity when interpreting physiological regions. Wavelet coefficients are obtained at each decomposition level and can be processed with different filtering procedures. We thus aim to obtain optimal results with minimal computational effort. This fact helps both the doctor and the patient.

Nowadays, medical images are digital and can be affected by non-uniform exposure, which gives a variable light intensity and poor contrast. The result will be low-contrast images, which affect the diagnosis accuracy. The visual quality of such an image might be improved if we enhance the contrast, locally modifying the light-intensity values (a procedure called contrast enhancement). There are several contrast-enhancement (CE) methods, such as histogram-based methods [20,21], including contrast-limited adaptive histogram equalization (CLAHE) [20]; homomorphic filters in the frequency-domain-based methods [22,23]; and non-linear quadratic-filter-based methods [23,24,25,26]. Multiscale-based CE techniques, such as those based on wavelets, outperform more conventional techniques (such as histogram-based methods) as the original signal can be viewed at several scales, stretching and dilating a basis function, the mother wavelet. This is a reason for choosing wavelets for CE, as their simplicity and mode of operation are more appropriate for real-life conditions. 

This study aims to apply a fast and simple CE algorithm that uses the dual-tree complex wavelet transform (DT-CWT) [24], based on adaptive filtering of wavelet coefficients and their selective amplification. The DT-CWT is a quasi-shift-invariant transform (a characteristic to be aimed at in the reconstruction of medical images) and has six orientations [24,25,26,27]. 

**Methods**: The proposed CE method acts in two steps. In the first step, the acquired image is denoised with the aid of a maximum a posteriori (MAP) filter applied in the DT-CWT domain. In the second step, the pixels of the result of the first step are nonlinearly amplified to enhance the contrast. The noise amplification in homogeneous areas is thus avoided by denoising the acquired image using the MAP filter. To increase the contrast of low-dynamic images, it is necessary to increase the apparition’s frequency of middle- and high-intensity pixels and to decrease the apparition’s frequency of low-intensity pixels. Angiogram images being mostly black-and-white, we will work only on the V component of the hue–saturation–value space to reduce computational effort. We will use the entire [0, 1] range, using the normalized V channel values [23].

*Dual-Tree Complex Wavelet Transform*: DT-CWT enables us to distinguish between positive and negative orientations, allowing selective processing of data, as it uses an analysis filter for the first level of decomposition and a different filter for the subsequent levels [28,29]. This property helps us to obtain an improved directional selectivity versus the discrete wavelet transform (DWT) and to adapt to the local level of clarity. As mentioned earlier, there are six types of subbands for the wavelet coefficients. Another advantage of using DT-CWT is its property of shift-invariance (a desirable property for medical images, which we aim to obtain after processing accurate, undistorted shapes). For the computation of DT-CWT, we used Professor Selesnick’s Matlab code, which can be found in [30].

The details of the steps of the algorithm already mentioned are presented as follows:

*Filtering (Denoising)*: Mathematical models are our way of explaining the observed facts. Statistical methods assume the wavelet coefficients are more likely to be modeled with a random signal with a certain distribution than the pixels of the image to be processed. This is one of the reasons why we apply statistical image processing methods in the wavelet domain. If the wavelet coefficients’ distribution is known, it is an easy task to filter out the unwanted artifacts with the aid of a MAP filter. As it is unknown, we assume an inter-dependency between the wavelet coefficients as a connection between the parent coefficients (with a given localization from a previous decomposition level) and children coefficients (with the same localization from the current decomposition level). As previous works about denoising/filtering proved their suitability [31,32], we use the bivariate Laplace’s distribution for the detail wavelet coefficients of the noiseless component of the acquired image and the bivariate normal distribution for the detail wavelet coefficients of the noise component of the acquired image for filtering with the aid of a MAP filter (called bishrink) in the DT-CWT domain. The noiseless coefficients’ distribution is shown in the next equation:

(2) $$p_{w}(w_{j},\;w_{j+1})=\frac{3}{2\pi \sigma^{2}}\exp\left(-\frac{\sqrt{3}}{\sigma }\sqrt{w_{j}^{2}+w_{j+1}^{2}}\right),$$

where *w* denotes the vector of parent and children wavelet coefficients, *w_j_* represents the children wavelet coefficients at the *^j^*^th^ decomposition level, and *w_j_*_+1_ represents the corresponding parent wavelet coefficients. The noisy coefficient distribution assumed in the case of the bishrink filter is a bivariate normal distribution (additive white Gaussian noise (AWGN)). We supposed that the noiseless pixels of the acquired image are additively perturbed by noise. Applying the DT-CWT (which is a linear transform) to the acquired image, whose pixels are denoted as *x_j_* in Equation (3), we obtain a mixture of two random processes: the sum of a bivariate Laplace distributed random process (representing the DT-CWT coefficients of the noise-free pixels and denoted as *w_j_* in Equation (3)) and a bivariate AWGN (representing the DT-CWT coefficients of the noisy pixels and denoted as *n_j_* in Equation (3)). We will use 6 decomposition levels for DT-CWT computation to reach an equilibrium between computational effort (which increases with the number of decompositions) and a sufficient number of iterations to remove the noisiest coefficients (Equation (3)):

*x_j_* = *w_j_* + *n_j_*. (3)

The proposed bishrink filter combines statistical models to obtain noise-free cardiac images, helping the physician with an early diagnosis. The input–output relation of the bishrink filter is the following:

(4) $${\hat{w}}_{j}=\frac{{\left(\sqrt{x_{j}^{2}+x_{j+1}^{2}}-\frac{\sqrt{3}\sigma_{n}^{2}}{\sigma }\right)}_{+}}{\sqrt{x_{j}^{2}+x_{j+1}^{2}}}x_{j},$$

where $x_{j+1}$ represents the value of the detail wavelet coefficient of the acquired image (with the same geometrical coordinates) at the following resolution level, (*j* + 1); σ is the local standard deviation of the wavelet transform of the noiseless component of the input image; and $\sigma_{n}^{2}$ is the noise variance. In practice, these last two parameters of the bishrink filter must be locally estimated.

### Local Contrast Enhancement

We adopted the method proposed in [23]. First, we need to define a contrast measure in the wavelet domain. A robust method is to define the ratio between the maximal value of the local standard deviation (computed in a moving window of size 7 × 7 centered on the current pixel $x_{j}$) and the current value of local standard deviation σ$\left(x_{j}\right)$ (Equation (5)):

(5) $$C\left(x_{j}\right)=\frac{max\left\{\sigma \left(x_{j}\right)\right\}}{\sigma \left(x_{j}\right)}.$$

The local contrast enhancement is performed next using these contrast values with the aid of an exponential function of the following form:

(6) $$A_{c}\left(x_{j}\right)=\exp\left(-\frac{1}{C\left(x_{j}\right)}\right)+A_{0},$$

where *A_C_* is a local amplification function and $A_{0}=1-exp(-1)$ is a normalization constant. So, the DT-CWT coefficients are filtered, and their local contrast is improved at the same time, as shown in Equation (7):

(7) $${\hat{w}}_{j}=A_{c}\left(x_{j}\right)bishrink\left(x_{j}\right)x_{j},$$

where *A_C_* is the local amplification in Equation (6) used for local contrast enhancement and *bishrink* is the MAP filter in (4) used for denoising. 

**Figure 3:**
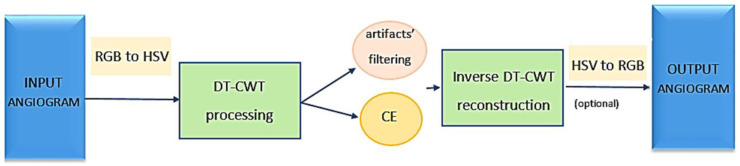
General view and data flow of the proposed wavelet algorithm.

The working flow of the algorithm for reducing the need for contrast substances (usually undesirable for newborns) is shown in Figure 3. 

Color images are generally represented in the red–green–blue (RGB) space. Still, we may work in the hue–saturation–value (HSV) space, and more specifically, only on the V channel (considering that most angiogram images are black and white), reducing the computational cost. The second step (not represented in Figure 3) is to normalize the V channel values to use the entire [0, 1] range. Next follows the processing into the DT-WT domain, consisting of denoising (artifact filtering) and local contrast enhancement (CE). The fourth step (not shown in Figure 3) is a de-normalization (to invert the second step) to realize a compression of the image’s dynamic. Next, the DT-CWT is inverted, and the resulting image is converted from HSV to RGB if the original angiogram is a color image.

**Materials:** The proposed method was applied to actual data from a public database [https://archive.physionet.org/physiobank/database/images/, accessed on 24 August 2023], so other scientists can also compare the results. The advantage of public databases over particular ones is that all scientists in the field domain may check the results and compare them with the results of their methods. We have processed several MRAs to visualize the fragile cardiopulmonary vasculature. These images were obtained in a study focused on coronal successive slices (slice thickness: 1.2 mm). These images were acquired through anteroposterior positions within the torso with a General Electric Signa 1.5 T imaging system. Artifacts are still present, although notable contrast substances, such as gadolinium contrast enhancement for visualization of the cardiac vasculature, have been used [32]. 

## 3. Results

The proposed algorithm was tested on data collected by the Massachusetts Institute of Technology (MIT) and publicly available online [33,34] to enable viable comparisons between software developers. For the DT-CWT transform, we used six decomposition levels, the Anton B filter, and a moving window size of 7 × 7 (as other studies have shown to give an estimate of the marginal standard deviation σ and noise variance $\sigma_{n}$). The following tables emphasize parameters such as the minimal and maximal local contrast (computed using Equation (5)) of the original images and the treated images. They aim to give insight into how these images can be perceived. We notice the small local contrast differences in the original images (Table 1a), implying smoothed images, with no notable differences between the different physiological areas. The mean contrast of the original images (Table 1b) has quite small values, with no significant differences between the areas of the original image, implying a poor outline of the images’ features.

To evaluate the performance of the wavelet-based CE method, we compare the results obtained with the CLAHE CE method (which is one of the state-of-the-art CE methods) for the treatment of the several (to avoid redundancy, but to show the algorithm’s performance, we included some of the results in Supplementary Materials) angiograms already considered. Table 2 shows the results of applying the CLAHE CE method to the ten angiograms. We notice significant differences between the images’ minimal and maximal local contrast values, implying a better outline of the different physiological areas scanned (Table 2a). The mean local contrast also reveals a high value (Table 2b). Thus, the contrast of the angiograms has been improved by applying the CLAHE method. 

**Figure 4:**
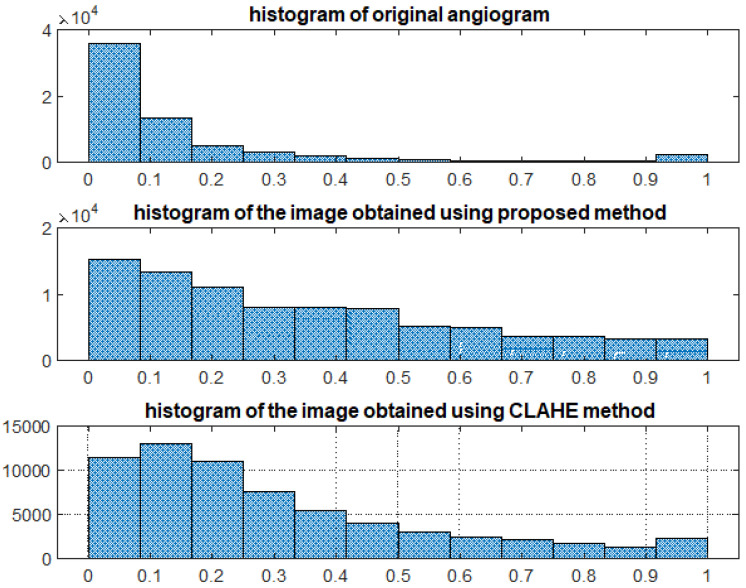
The variability of the data displayed in Table 1, Table 2 and Table 3 shows greater stability for the wavelet-based CE method, with little deviations, implying that this method can be used to find the optimal way to treat all types of underexposed angiograms.

Lastly, Table 3a,b show the changes in the same angiograms obtained by applying the wavelet-based CE method. At first perusal, we notice that, as in the case of the treatment with the CLAHE method, significant differences appear between the minimal and maximal local contrast values, implying a better outline of the different physiological areas scanned as in the case of the original images (Table 1a). The mean local contrast also reveals large values (Table 3b). Hence, the DT-CWT-based CE method dramatically improves the contrast performance of angiograms. Not only has the contrast of original images been enhanced using the DT-CWT-based CE method, but the results obtained are comparable to or better than those obtained by applying state-of-the-art CE methods, such as the CLAHE method. Compared to the CLAHE method, the values obtained using the DT-CWT-based CE method are better grouped, suggesting a more uniform treatment. This remark is confirmed by the graphs in Figure 4. Indeed, the most uniform (horizontal) graph in Figure 4 corresponds to the wavelet-based CE method. So, using this method, we obtain the better equalization of low-, medium-, and high-intensity pixels in the angiograms.

These objective parameters should be reflected when we display the studied images graphically and help the physician perform correct diagnoses (with no physiological features hidden in low-contrast zones). We hope it will be accurate enough to avoid the need for contrast substances and avoid possible unnecessary complications.

**Figure 5:**
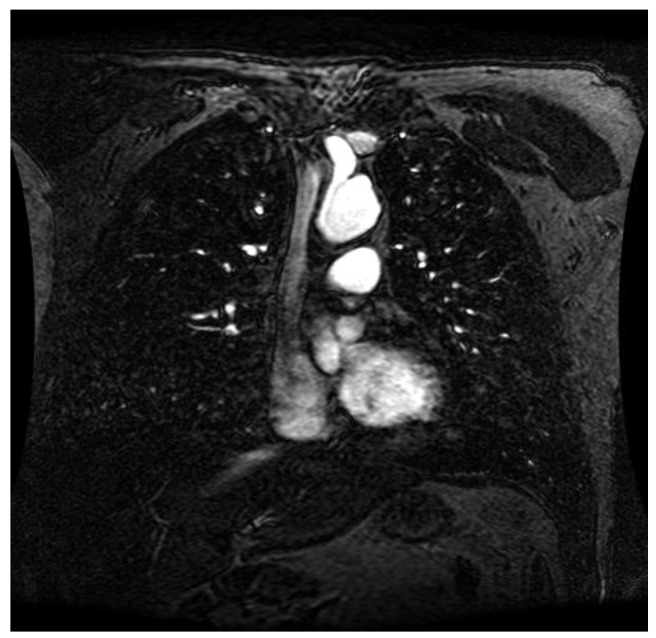
Original angiogram E1154S7I064. Despite the use of contrast substances (gadolinium contrast enhancement), the visibility is reduced.

**Figure 6:**
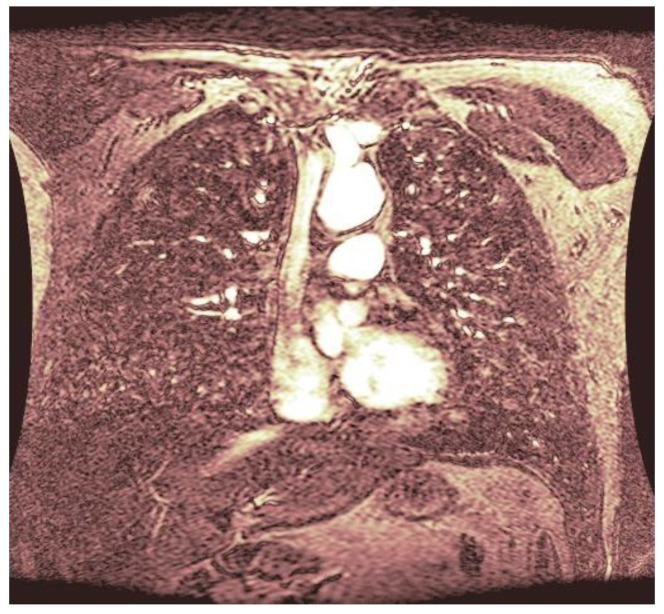
The image was obtained by denoising the angiogram E1154S7I064 using the CLAHE method. The contrast is already improved, but the whole areas of the image are too luminous, details might escape.

**Figure 7:**
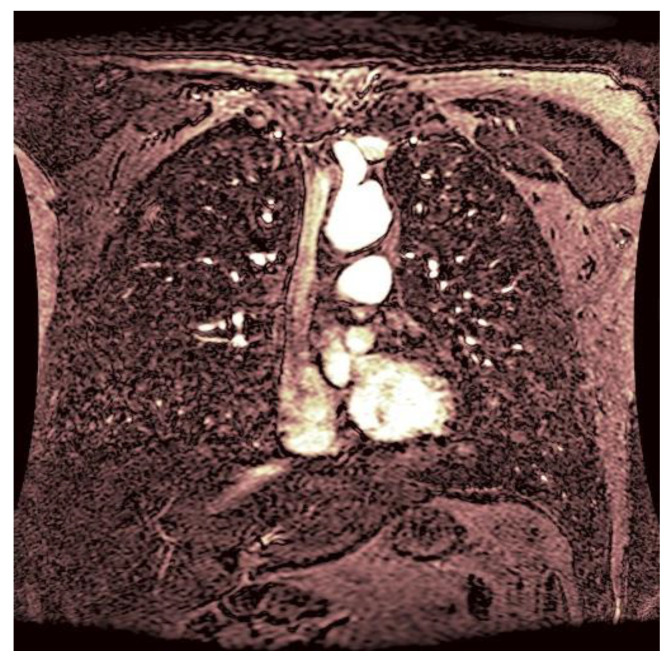
The result of the wavelet-based CE method applied to the angiogram E1154S7I064 with visible physiological features to improve diagnosis.

**Figure 8:**
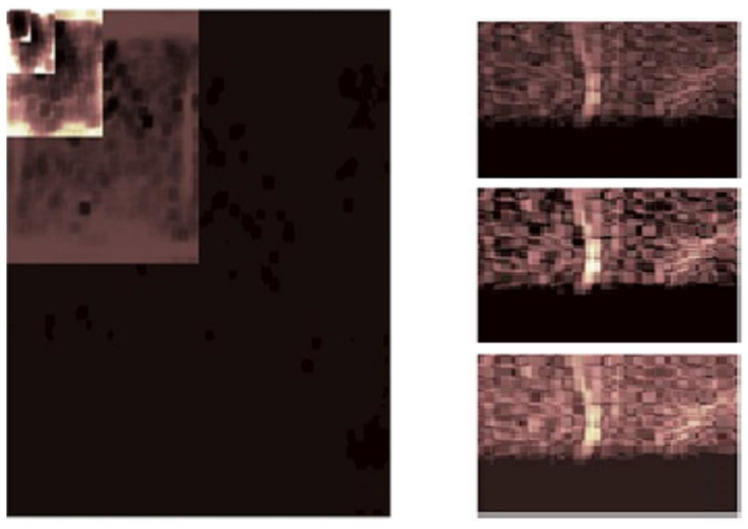
Different results to show the contrast improvements given by different CE algorithms. Left image: local contrast of the absolute value of the DT-CWT image corresponding to the angiogram E1154S7I064. Right images: zoom in the same region of the original image (**up**), result of wavelet-based CE method (**middle**), and result of CLAHE method (**bottom**).

**Figure 9:**
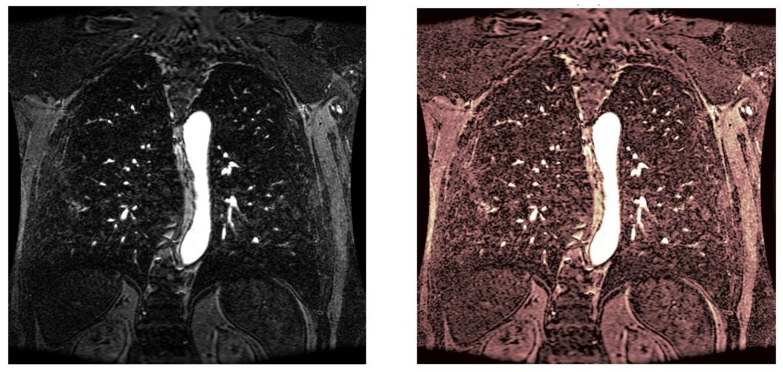
Original angiogram E1154S7I001 (**left**) and wavelet-based CE method result (**right**).

**Figure 10:**
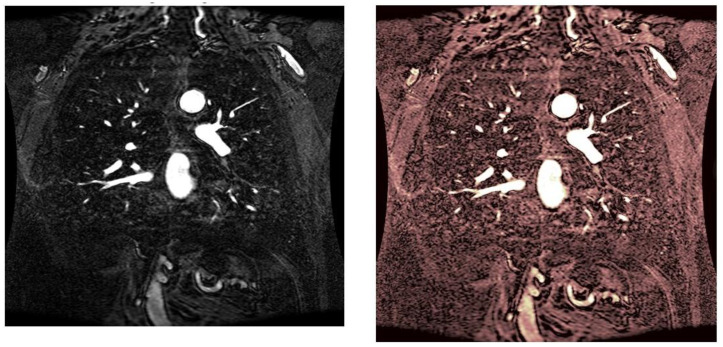
Original angiogram E1154S7I026 (**left**) and wavelet-based CE method result (**right**).

**Figure 11:**
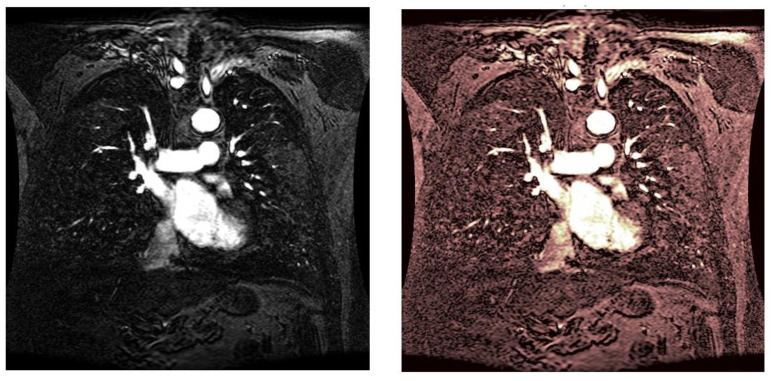
Original angiogram E1154S7I053 (**left**) and wavelet-based CE method result (**right**).

**Figure 12:**
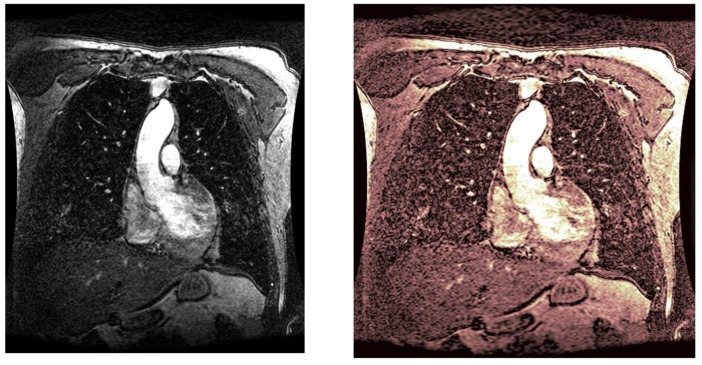
Original angiogram E1154S7I075 (**left**) and wavelet-based CE method result (**right**).

As statistical data have less relevance for life sciences, we add graphical displays of the studied angiograms. Surprisingly, even a simple denoising procedure can help reduce unwanted artifacts, as can be observed when comparing the angiogram E1154S7I064 (shown in Figure 5) with the result obtained by denoising it with the CLAHE method already described (shown in Figure 6). The corresponding image obtained by the wavelet-based CE method (shown in Figure 7) better outlines the biological features, making visible some details that cannot be observed in the original image (Figure 5), and allows us to take into consideration relinquishing contrast substances (a fact with multiple benefits for newborns and children). A visual comparison of CLAHE and wavelet-based CE method results in the performance obtained for this angiogram can be made with the images in Figure 8. It can be noticed that the original image is too dark to observe all the significant medical details and that the result of CLAHE is too luminous and can mask medical features. The same remarks concerning the advantages of the wavelet-based CE methods can be made upon a visual analysis of the original angiograms and the corresponding results shown in Figure 9, Figure 10, Figure 11 and Figure 12.

## 4. Discussion

Research in the field of medical imaging for the treatment of heart diseases is growing exponentially. Unfortunately, more and more cardiac problems befall us at a young age (for example, the SARS-CoV-2 pandemic is primarily associated with cardiac issues, which we are only slowly understanding nowadays). These problems are harder to prevent because of patients’ stressful lives. Problems pass unnoticed until they cannot be ignored, and untreated symptoms may lead to notable cardiac malfunctions. Sometimes the contrast of cardiovascular images is not satisfactory, even if contrast-enhancing substances are used for the acquisition. Important details can be revealed if these images are processed with contrast improvement algorithms. The use of contrast enhancement algorithms becomes even more interesting if this technique can lead to the elimination of the need for contrast agents for image acquisition. We aim to help physicians to enhance the contrast and use as few harmful chemicals as possible. If an ultrasound image can be improved through efficient and cost-effective software, then hospitals, adults, and pediatric sections should have access to it.

We have chosen to use the DT-CWT for CE. This tool has already shown its efficiency for medical image enhancement [35,36]. In [35], an efficient medical image fusion system based on DT-CWT and the modified central force optimization (MCFO) technique is presented. Simulation results demonstrate that the proposed optimized DT-CWT medical image fusion system based on MCFO and histogram matching achieves a superior performance with better image quality and much more detail. In [36], a different DT-CWT-based CE method is presented. As the authors of [36] appreciate, enhancing the quality of diagnostic images and preserving their original features is crucial for early detection and further analysis. In non-contrast CT imaging, a noisy and low-contrast CT image can give inadequate information for the visual analysis of affected regions. A new method for enhancing non-contrast CT images with DT-CWT and adaptable morphology is presented in [36]. Input CT images are inserted into the DT-CWT system, resulting in low- and high-frequency subbands. On high-frequency subbands, denoising is performed using the wavelet-related shearlet transform method, which results in enhanced high-frequency sub-images. An adaptive morphology top-hat transform technique is used with DCT-based local enhancement to increase the quality of low-frequency sub-images. The improved low- and high-frequency components are then recombined to form the enhanced CT image using inverse DT-CWT. In order to estimate the success of the proposed system, experiments and validations were carried out by the authors of [36] on a diverse collection of CT images taken from publicly accessible databases. An extensive quantitative analysis demonstrates that the proposed method outperforms existing image enhancement techniques in terms of peak signal-to-noise ratio, entropy, contrast ratio, and measure of enhancement. In the proposed algorithm, the contrast is enhanced while the brightness and natural characteristics of the CT image are maintained.

We propose a DT-CWT-based CE method in two steps (simple filtering and statistical local CE method) to improve the contrast of angiograms. It performs similarly to the method presented in [24] but is faster, allowing a more significant number of MRA images to be processed in the same time. Both methods use the DT-CWT, but the bishrink filter is faster than the filter used in [24]. Both methods are faster than artificial intelligence (AI)-based CE methods because they do not use neural networks and because the decomposition scheme implemented for wavelet transforms is very fast.

The algorithms’ performance has also been evaluated using several parameters. The objective parameters considered in this paper, namely the contrast data in Table 1, Table 2 and Table 3 and the histograms (Figure 4), show the superiority of the wavelet-based CE algorithm compared to the CLAHE CE method.

To help physicians, we displayed the results for randomly selected figures (Figure 5, Figure 6, Figure 7, Figure 8, Figure 9, Figure 10, Figure 11 and Figure 12), in the same way the ultrasound images are interpreted by a physician, to facilitate diagnosis in a clinical environment. We believe the new images better outline any cardiac abnormalities.

## 5. Conclusions

This study was initiated to reduce unnecessary stress and pain caused by an unsecured or even by an incorrect diagnosis made by less experienced physicians, due to lack of details in images. We, as engineers, want to help avoid such situations. Thus, we have developed a two-step wavelet-based algorithm for the CE of angiograms, involving the filtering of the original image and then the efficient processing of the remaining coefficients. To preserve the computational simplicity of the task, we estimated a reduced number of parameters. We also applied a simplified filtering procedure based on a simple statistical model (the Laplace distribution).

We obtained accurate results (displayed graphically in Figure 5, Figure 6, Figure 7, Figure 8, Figure 9, Figure 10, Figure 11 and Figure 12 and quantitatively in Table 1, Table 2 and Table 3 and Figure 4). The contrast values for the original image are small, indicating that there is a poor contrast in the original angiogram and thus a poor outline of the image’s features. The contrast values are higher after using contrast enhancement software procedures, such as the CLAHE method or the proposed wavelet-based method. Both CE methods show a good global contrast, but the DT-CWT-based method has greater stability, the values not depending too closely on the original state of the acquired MRA image. The CLAHE method has larger variation in the values (mean global contrast between 1.1183 × 10^4^ and 2.4971 × 10^4^). Thus, the performance might depend on the acquisition factors. The wavelet algorithm displays less variation and shows greater stability. Thus, it does not need further adjustments to be applied on a large number of MRA images, to enhance their contrast. Applying the DT-CWT method, we obtain a better distribution of the low-, medium-, and high-intensity pixels in the angiograms. The results are outlined in several figures for randomly selected MRA images (Figure 5, Figure 6, Figure 7, Figure 8, Figure 9, Figure 10, Figure 11 and Figure 12)—there are more image features/details to be noticed after applying the contrast enhancement based on the DT-CWT method.

The performance of the proposed method is similar to the performance of other resource-consuming CE algorithms, such as CLAHE (Figure 4), but the wavelet-based CE method adopted is faster than CLAHE or the one presented in [23]. Using simpler estimation methods, we have simplified the filtering procedure, requiring the estimation of a reduced number of parameters and demanding low computational effort.

This method works well for dark images when exposure conditions are limited.

The developed algorithm is part of a bigger project, a pilot study at this moment, to see its performance. We will include pathological evidence in future works, as we will closely collaborate with physicians. The physicians will observe and describe all lesions observed in the MRA images in the lungs, heart, and other internal organs. 

Future work will involve collaborating with physicians from the Medicine and Pharmacy Faculty Timisoara (UMFT)—the *Neonatology Section*—to test and optimize the proposed algorithm on newborns and children (mother wavelet, number of decomposition levels, and other statistical distribution probably better suited for children). We aim to avoid, as much as possible, the use of contrast substances in ultrasound, computed tomography, or magnetic resonance techniques. In this direction, we will use the DT-CWT-based CE method in an effort to improve the contrast of time-of-flight (ToF) angiograms [37], which are MR images acquired without the injection of any contrast substance.

## Figures and Tables

**Table 1:** (**a**) Statistical data for envisioning the local contrast of the original images. (**b**) Statistical data for envisioning the global contrast of the original image.

(a)		
Angiogram	Minimal Local Contrast	Maximal Local Contrast
E1154S7I001	0.8323	643.1550
E1154S7I026	0.4780	1.1602 × 10^3^
E1154S7I053	0.5058	1.4905 × 10^3^
E1154S71064	0.6603	726.4346
E1154S7I075	0.8827	613.5881
**(b)**		
**Angiogram**	**Mean Contrast**	
E1154S7I001	7.7508	
E1154S7I043	11.5843	
E1154S7I053	6.8846	
E1154S71064	7.4985	
E1154S7I075	16.9591	

**Table 2:** (**a**) Statistical data for envisioning the local contrast software changes obtained with the CLAHE method. (**b**) Statistical data for envisioning the global contrast software changes obtained with the CLAHE method.

(a)		
Angiogram	Minimal Local Contrast	Maximal Local Contrast
E1154S7I001	1.00	1.8627 × 10^6^
E1154S7I026	1.00	1.4606 × 10^6^
E1154S7I053	1.00	2.0907 × 10^6^
E1154S71064	1.00	2.8498 × 10^6^
E1154S7I075	1.00	1.5664 × 10^6^
**(b)**		
**Angiogram**	**Mean Contrast**	
E1154S7I001	2.4315 × 10^4^	
E1154S7I026	1.0466 × 10^4^	
E1154S7I053	1.1183 × 10^4^	
E1154S71064	2.4971 × 10^4^	
E1154S7I075	1.3194 × 10^4^	

**Table 3:** (**a**) Statistical data for envisioning the local contrast software changes obtained with the DT-CWT-based method. (**b**) Statistical data for envisioning the global contrast software changes obtained with the DT-CWT-based method.

(a)		
Angiogram	Minimal Local Contrast	Maximal Local Contrast
E1154S7I001	1.00	1.8548 × 10^6^
E1154S7I026	1.00	2.4205 × 10^6^
E1154S7I053	1.00	1.7657 × 10^6^
E1154S71064	1.00	1.8979 × 10^6^
E1154S7I075	1.00	2.5428 × 10^6^
**(b)**		
**Angiogram**	**Mean Contrast**	
E1154S7I001	2.4306 × 10^4^	
E1154S7I026	2.4022 × 10^3^	
E1154S7I053	2.4238 × 10^3^	
E1154S71064	1.8504 × 10^4^	
E1154S7I075	2.2667 × 10^4^	

## Data Availability

The analyzed data supporting this study can be found at https://physionet.org/content/?topic=mra (accessed on 24 August 2023).

## Supplementary Materials

The following supporting information can be downloaded at: https://www.mdpi.com/article/10.3390/life13112160/s1.
