# Supplementary material for: Improving Chest Monitoring through Magnetic Resonance Angiogram Image Contrast Enhancement
Source: Life (Basel). 2023 Nov 3;13(11):2160. doi: 10.3390/life13112160 (PMC10672579; doi:10.3390/life13112160)
Supplement: Supplementary file 1 [file life-13-02160-s001.zip › life-2618252-supplementary.pdf]

Supplementary materials

RANDOMLY SELECTED AND FILTERED MRA (MIT Database) IMAGES

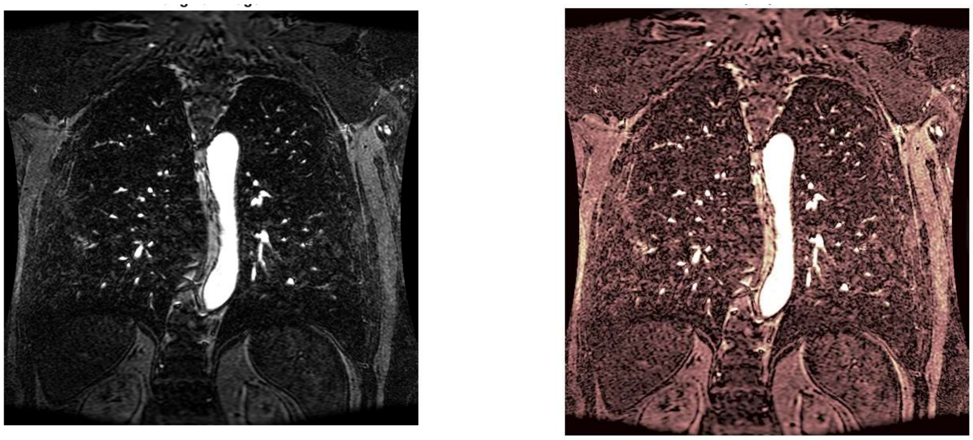

Figure S1. Original angiogram E1154S7I001 (left) and wavelet-based CE method result (right).

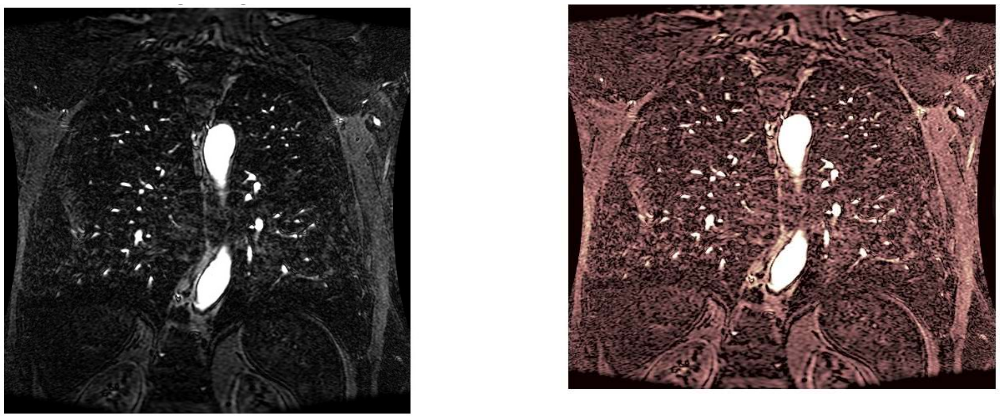

Figure S2. Original angiogram E1154S7I004 (left) and wavelet-based CE method result (right).

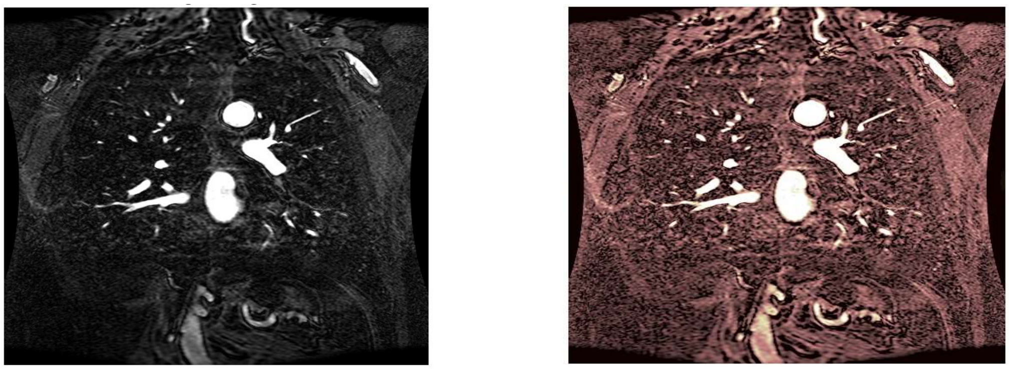

Figure S3. Original angiogram E1154S7I026 (left) and wavelet-based CE method result (right).

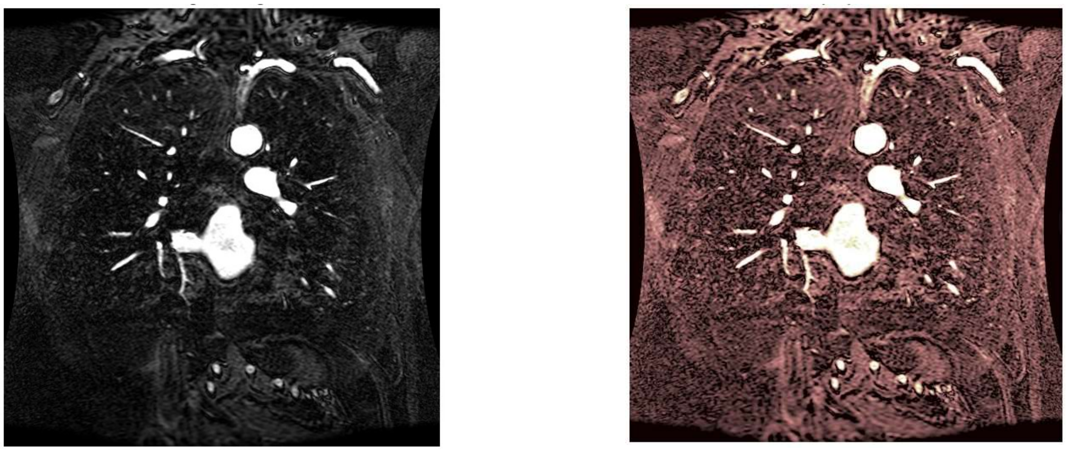

Figure S4. Original angiogram E1154S7I030 (left) and wavelet-based CE method result (right).

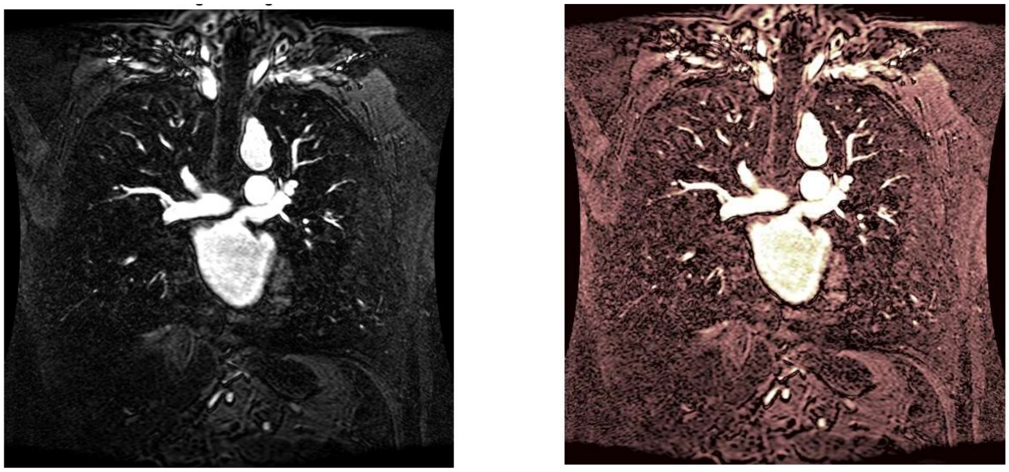

Figure S5. Original angiogram E1154S7I043 (left) and wavelet-based CE method result (right).

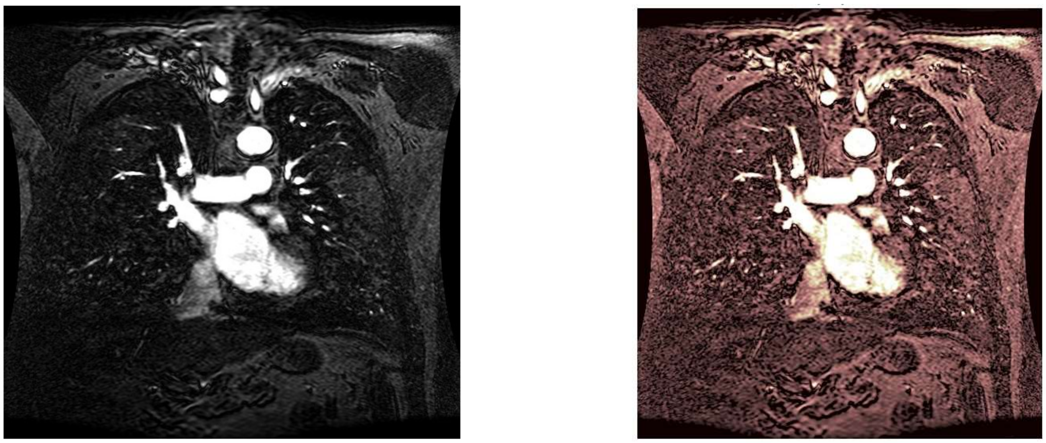

Figure S6. Original angiogram E1154S7I053 (left) and wavelet-based CE method result (right).

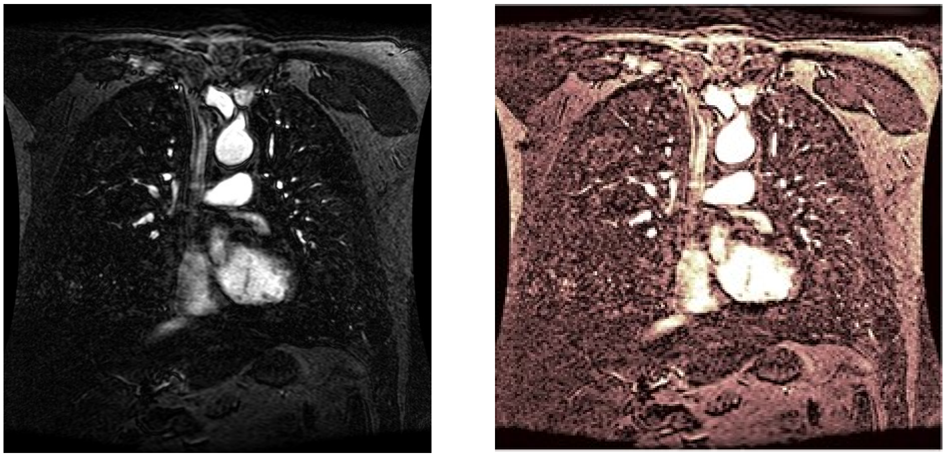

Figure S7. Original angiogram E1154S7I060 (left) and wavelet based CE method result (right).

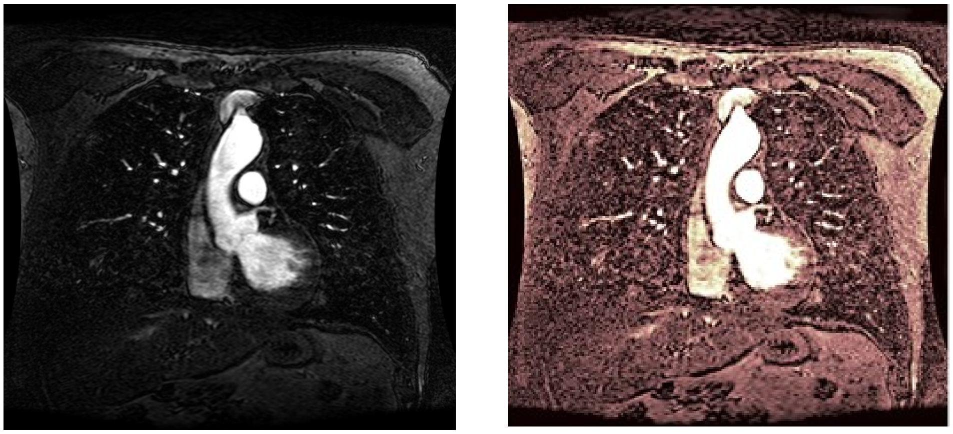

Figure S8. Original angiogram E1154S7I070 (left) and wavelet based CE method result (right).

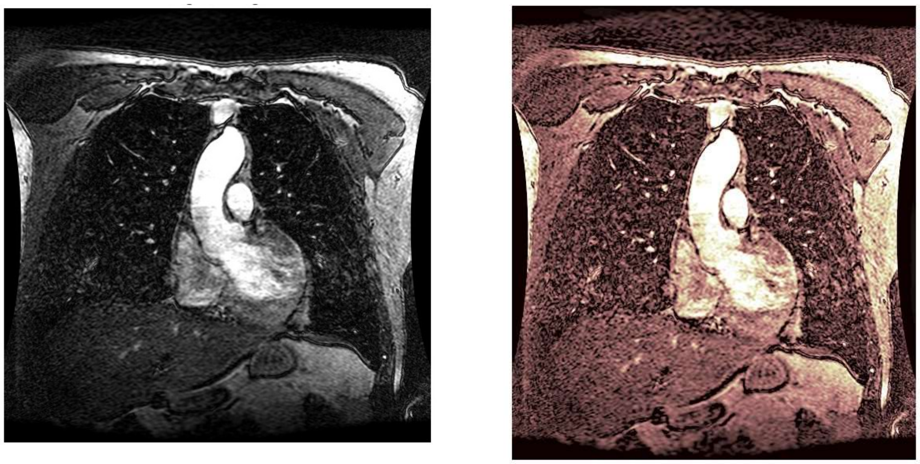

Figure S9. Original angiogram E1154S7I075 (left) and wavelet based CE method result (right).
